# Abnormal Cortical Networks in Mild Cognitive Impairment and Alzheimer's Disease

**DOI:** 10.1371/journal.pcbi.1001006

**Published:** 2010-11-18

**Authors:** Zhijun Yao, Yuanchao Zhang, Lei Lin, Yuan Zhou, Cunlu Xu, Tianzi Jiang, the Alzheimer's Disease Neuroimaging Initiative

**Affiliations:** 1 Center for Computational Medicine, School of Information Science and Engineering, Lanzhou University, Lanzhou, China; 2 LIAMA Center for Computational Medicine, National Laboratory of Pattern Recognition, Institute of Automation, Chinese Academy of Sciences, Beijing, China; 3 Key Laboratory for NeuroInformation of Ministry of Education, School of Life Science and Technology, University of Electronic Science and Technology of China, Chengdu, China; 4 Department of Mathematics, Zhejiang University, Hangzhou, China; 5 Center for Social and Economic Behavior, Institute of Psychology, Chinese Academy of Sciences, Beijing, China; University College London, United Kingdom

## Abstract

Recently, many researchers have used graph theory to study the aberrant brain structures in Alzheimer's disease (AD) and have made great progress. However, the characteristics of the cortical network in Mild Cognitive Impairment (MCI) are still largely unexplored. In this study, the gray matter volumes obtained from magnetic resonance imaging (MRI) for all brain regions except the cerebellum were parcellated into 90 areas using the automated anatomical labeling (AAL) template to construct cortical networks for 98 normal controls (NCs), 113 MCIs and 91 ADs. The measurements of the network properties were calculated for each of the three groups respectively. We found that all three cortical networks exhibited small-world properties and those strong interhemispheric correlations existed between bilaterally homologous regions. Among the three cortical networks, we found the greatest clustering coefficient and the longest absolute path length in AD, which might indicate that the organization of the cortical network was the least optimal in AD. The small-world measures of the MCI network exhibited intermediate values. This finding is logical given that MCI is considered to be the transitional stage between normal aging and AD. Out of all the between-group differences in the clustering coefficient and absolute path length, only the differences between the AD and normal control groups were statistically significant. Compared with the normal controls, the MCI and AD groups retained their hub regions in the frontal lobe but showed a loss of hub regions in the temporal lobe. In addition, altered interregional correlations were detected in the parahippocampus gyrus, medial temporal lobe, cingulum, fusiform, medial frontal lobe, and orbital frontal gyrus in groups with MCI and AD. Similar to previous studies of functional connectivity, we also revealed increased interregional correlations within the local brain lobes and disrupted long distance interregional correlations in groups with MCI and AD.

## Author Summary

Understanding the progression of Alzheimer's disease (AD) is essential. We investigated networks of cortical connectivity along a continuum from normal to AD. Mild Cognitive Impairment (MCI) has been implicated as transitional between normal aging and AD. By investigating the characteristics of cortical networks in these three stages (normal, MCI and AD), we found that all three networks exhibited small-world properties. These properties indicate efficient information transfer in the human brain. We also found that the small-world measures of the MCI network were intermediate to those of the normal controls and the patients with AD. This supports the opinion that MCI is a transitional stage between normal aging and AD. Additionally, we found altered interregional correlations in patients with MCI and AD, which may indicate that a compensatory system interacts with cerebral atrophy. The presence of compensatory mechanisms in patients with MCI and AD may enable them to use additional cognitive resources to function on a more nearly normal level. In future, we need to integrate the multi-level network features obtained with various functional and anatomical brain imaging technologies on different scales to understand the pathophysiological mechanism of MCI and AD. We propose brainnetome to represent such integration framework.

## Introduction

Alzheimer's disease, the most common form of dementia, is associated with plaques and tangles in the brain which would lead to a loss of neurons and synapses [1]–[2]. In the early stages, Alzheimer's disease is characterized by a decline in cognitive and memory functions. Clinical symptoms of Alzheimer's disease include confusion, aggression, language breakdown, and the loss of cognitive functions [3]–[4]. Mild Cognitive Impairment (MCI), characterized by memory impairment, is considered to be the clinical transition stage between normal aging and dementia [5]–[6]. Studies suggest that subjects with MCI tend to progress to probable Alzheimer's disease at a rate of approximately 10% to 15% per year [7]. Facing these serious facts, many research groups have studied AD and MCI from various perspectives, attempting to understand the pathogenesis with a goal of discovering effective therapies [8]–[9]. Voxel based morphometry (VBM), proposed by Friston and Ashburner [10], allows a fully automated whole-brain analysis of structural MRI scans [11]. Using the VBM method, previous studies showed atrophy of the parahippocampal gyrus, medial temporal lobe [12], entorhinal cortex, cingulum [13], insula and thalamus [14] in subjects with MCI and atrophy of the entire hippocampus and some localized regions in the temporal lobe, cingulum, precuneus, insular cortex, caudate nucleus, and frontal cortex [14]–[16] in patients with AD.

Recently, studies of functional and structural brain networks in AD patients have indicated that cognitive function deficits could be due to abnormalities in the connectivity between different brain areas. These brain areas include the bilateral parietal regions, middle temporal gyrus, cingulum, medial frontal gyrus, precentral gyrus, fusiform, etc. [17]–[19]. Small-worldness, which was characterized by a high degree of clustering and short path lengths, has been found to exist in social networks, the connectivity of the internet, and in gene networks [20]–[21]. Previous studies have reported that the human cortical network also has small-world properties [22]–[25], and a loss of small-world characteristics has been detected in patients with AD [18]–[19]. Reports on the characteristics of the structural cortical network in MCI have been rare [26]. In the present study, we constructed structural cortical networks using average gray matter volumes of each AAL area to investigate the characteristics of the cortical networks in NCs, MCI subjects and AD patients. In addition, we also inspected the pattern of structural connections and hub regions. This type of research may contribute to understanding the pathogenesis of MCI and AD. Since MCI is considered to be an intermediate stage between normal aging and AD, we hypothesized that the measurements of the cortical network properties (for example clustering coefficient and absolute path length) in MCI would lie between those of NC and AD subjects.

## Results

**Figure 1:**
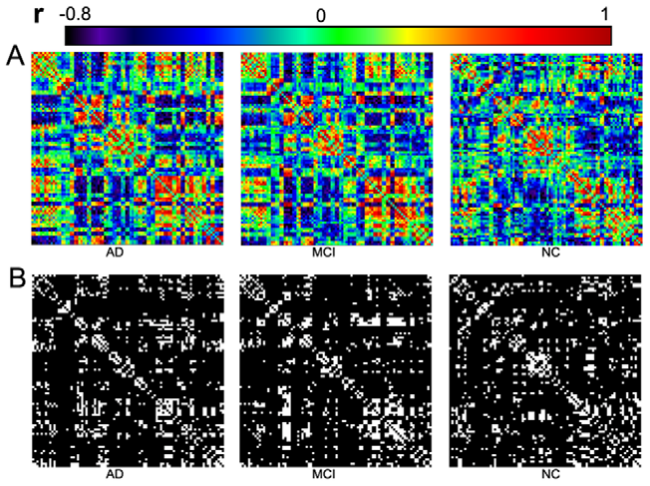
The interregional correlations matrix in the AD, MCI and NC groups. The color bar indicates the value of the correlation coefficient r (ranging from −0.8 to 1). **A**. The correlations matrices obtained by calculating the correlations between pairs of AAL areas within each group (left - the AD group, middle - the MCI group and right - the NC group). **B**. The binarized matrices obtained by thresholding the above correlations matrices of **A** with a sparsity threshold (15%). The sparsity threshold sets the same number of nodes and edges in each of the three cortical networks.

The interregional correlation coefficients of the cortical networks were calculated to construct correlation matrices (90×90) for the NC, MCI and AD groups (see Materials and Methods). The images of the interregional correlation matrices are shown in Figure 1. We revealed one feature in common among the three groups that strong interregional correlations exist between most homotopic regions (the same areas in opposite hemispheres). This finding is consistent with earlier studies using cortex thickness [24] and gray matter volume [27].

### Small-world properties of cortical networks

**Figure 2:**
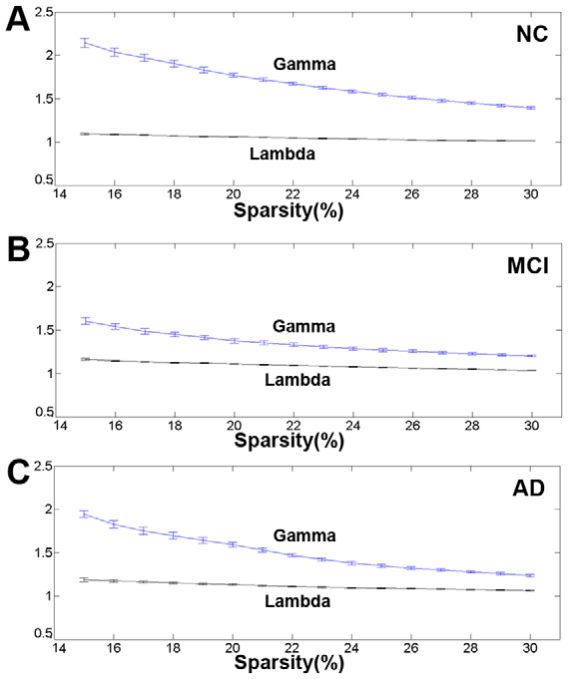
Small-world properties of the structural cortical networks. The graphs show the absolute path lengths (Gamma γ = C_p_^real^/C_p_^rand^) and clustering coefficients (Lambda λ = L_p_^real^/L_p_^rand^) over a wide range of sparsity values (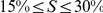) and the error bars were obtained using bootstrap method. All the networks have γ>1 (the blue lines) and λ≈1 (the black lines), which imply small-world properties. As the values of the sparsity thresholds increase, the γ values decrease rapidly and the λ values change only slightly. **A** – The values of Gamma and Lambda in NC. **B** – The values of Gamma and Lambda in MCI. **C** – The values of Gamma and Lambda in AD.

**Figure 3:**
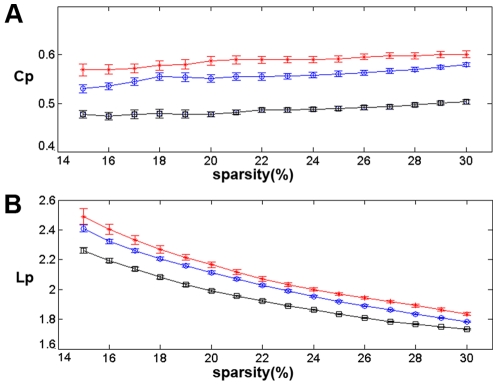
Mean clustering coefficients and mean absolute path lengths of the cortical networks in the three subject groups. Mean clustering coefficient (C_p_) and mean absolute path length (L_p_) over a wide range of sparsity values (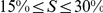) and the error bars were obtained using bootstrap method. **A** - The red stars represent the mean clustering coefficient in the AD group. The blue circles represent the mean clustering coefficient in the MCI group. The black squares represent the mean clustering coefficient in the NC group. **B** - The red stars represent the mean absolute path length in the AD group. The blue circles represent the mean absolute path length in the MCI group. The black squares represent the mean absolute path length in the NC group. The mean clustering coefficient was the greatest for the AD group and the absolute path length was shortest for the NC group. The measurements of the MCI group were intermediate between the NCs and ADs.

**Figure 4:**
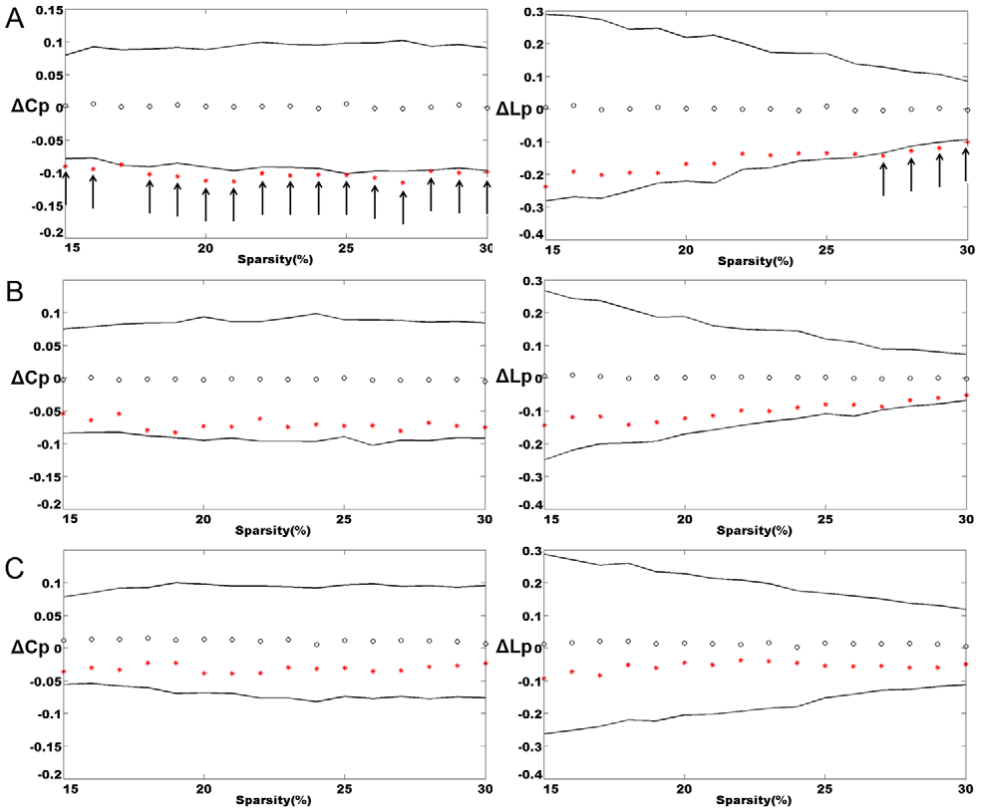
Between-group differences in the clustering coefficient (C_p_) and the absolute path length (L_p_) over a range of sparsity values. The left shows the between-group differences in clustering coefficients (ΔCp) and the right shows the between-group differences in absolute path lengths (ΔLp) over a wide range of sparsity values (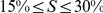). The black open circles represent the mean values and the black lines represent the 95% confidence intervals of the between-group differences obtained from 1000 permutation tests at each sparsity value. **A** - Differences between the NC and AD groups (ΔCp = Cp_NC_−Cp_AD_, ΔLp = Lp_NC_−Lp_AD_). **B** - Differences between the NC and MCI groups (ΔCp = Cp_NC_−Cp_MCI_, ΔLp = Lp_NC_−Lp_MCI_). **C** - Differences between the MCI and AD groups (ΔCp = Cp_MCI_−Cp_AD_, ΔLp = Lp_MCI_−Lp_AD_). The arrows indicate the significant (p<0.05) between-group differences in the clustering coefficients and absolute path lengths.

Some recent studies demonstrated that small-world properties are exhibited in functional brain networks [23], [28] and structural brain networks [24], [29]. Compared with random networks, small-world networks have higher clustering coefficients and similar shortest absolute path length. Over a wide range of sparsity values (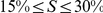), clustering coefficients and absolute path lengths were calculated for the three networks. The small-world attributes of three cortical networks are shown in Figure 2. Compared with matched random networks which have the same number of nodes and degree distribution, the three cortical networks had similarly identical absolute path lengths (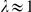) and larger clustering coefficients (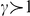) (see Materials and Methods). A precise quantitative analysis suggests that small-world networks with a high global efficiency and an optimal organization can support distributed information processing and high dynamic complexity [25]. Similar to previous studies, the cortical networks of the groups with MCI and AD showed varying degrees of loss of small-world characteristics [18]–[19]. As shown in Figure 3, the clustering coefficient was the greatest for the AD group and the absolute path length was shortest for the normal controls. Additionally, the corresponding measurements were intermediate for the MCI group among the three cortical networks. A permutation test was used to detect the statistical significance of the between-group differences of the attributes (see Materials and Methods). In Figure 4, the arrows indicated the significant differences between NCs and ADs in the cluster coefficients (p<0.05) at most of the sparsity values. The differences between NCs and ADs in the absolute path lengths were significant at higher sparsity values (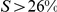). And we detected no significant differences in the clustering coefficients and the absolute path lengths between the NC and MCI groups and between the MCI and AD groups (p>0.05). Our findings provided additional support for the hypothesis that the cortical networks had a further loss in the small-world characteristics in the progression from MCI to AD [18]–[19].

### Measurements of the cortical networks

In order to detect the specific between-group differences among the three cortical networks, a fixed sparsity threshold value (sp = 15%) was used. This sparsity value can ensure that the cortical networks are fully connected while minimizing the number of false-positive paths [19], [23].

#### Hub regions of the three cortical networks

To ascertain the hub regions of the cortical networks, the normalized betweenness centrality (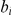) of each node was evaluated (see Materials and Methods). Hub nodes were defined as those whose betweenness values were more than twice the average betweenness of the network (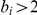). Based on our results, some regions were identified as hub regions in the cortical networks of each of the three populations. Details of the hub regions in the three cortical networks are shown in Table 1.

**Table 1:** Hub regions in cortical networks of the three populations listed in descending order of their normalized betweenness in the NCs.

AAL areas	Betweenness		
	NC	MCI	AD
Temporal_Mid_R	4.660	1.297	0.106
Lingual_R	3.474	2.793	0
Frontal_Med_Orb_R	2.898	7.341	1.116
Lingual_L	2.825	0.133	0
Paracentral_Lobule_R	2.641	1.830	3.806
Frontal_Mid_Orb_L	2.601	0.284	0
Frontal_Med_Orb_L	2.575	1.382	2.253
Occipital_Mid_L	2.462	3.819	2.194
Temporal_Pole_Sup_L	2.287	0.629	0.332
Parietal_Sup_R	2.256	0.623	0.522
Paracentral_Lobule_L	2.253	2.935	3.069
Frontal_Inf_Orb_L	2.237	1.579	1.697
ParaHippocampal_R	2.084	0.984	1.649
Insula_L	2.049	0.240	1.038
Parietal_Sup_L	1.782	3.095	2.544
Caudate_R	1.632	3.8108	0.036
Putamen_R	1.207	0	3.189
Precuneus_L	1.034	3.612	5.974
Temporal_Sup_R	1.025	2.176	1.428
Frontal_Inf_Orb_R	1.022	2.713	4.494
Frontal_Inf_Tri_R	0.975	2.159	2.442
Postcentral_R	0.963	2.026	0.964
Cingulum_Ant_R	0.652	0.178	2.367
Cuneus_L	0.639	2.534	5.534
Precentral_R	0.547	2.827	2.612
Insula_R	0.529	1.741	3.321
Supp_Motor_Area_R	0.358	2.489	0.958
Rolandic_Oper_R	0.329	1.094	6.464
Pallidum_R	0.165	4.122	0
Calcarine_L	0.125	0.761	3.864

**Table 2:** The abbreviations of AAL regions except the cerebellum.

Region ID	AAL Regions	Abbreviation
1	Precentral	PreCG
2	Frontal_Sup	SFGdor
3	Frontal_Sup_Orb	ORBsup
4	Frontal_Mid	MFG
5	Frontal_Mid_Orb	ORBmid
6	Frontal_Inf_Oper	IFGoperc
7	Frontal_Inf_Tri	IFGtriang
8	Frontal_Inf_Orb	ORBinf
9	Rolandic_Oper	ROL
10	Supp_Motor_Area	SMA
11	Olfactory	OLF
12	Frontal_Sup_Medial	SFGmed
13	Frontal_Med_Orb	ORBsupmed
14	Rectus	REC
15	Insula	INS
16	Cingulum_Ant	ACG
17	Cingulum_Mid	DCG
18	Cingulum_Post	PCG
19	Hippocampus	HIP
20	ParaHippocampal	PHG
21	Amygdala	AMYG
22	Calcarine	CAL
23	Cuneus	CUN
24	Lingual	LING
25	Occipital_Sup	SOG
26	Occipital_Mid	MOG
27	Occipital_Inf	IOG
28	Fusiform	FFG
29	Postcentral	PoCG
30	Parietal_Sup	SPG
31	Parietal_Inf	IPL
32	SupraMarginal	SMG
33	Angular	ANG
34	Precuneus	PCUN
35	Paracentral_Lobule	PCL
36	Caudate	CAU
37	Putamen	PUT
38	Pallidum	PAL
39	Thalamus	THA
40	Heschl	HES
41	Temporal_Sup	STG
42	Temporal_Pole_Sup	TPOsup
43	Temporal_Mid	MTG
44	Temporal_Pole_Mid	TPOmid
45	Temporal_Inf	ITG

In our work, the identified hub regions involved the middle temporal gyrus, temporal pole, lingual gyrus, orbital frontal gyrus, and superior parietal gyrus in the NC group and the orbital frontal gyrus, inferior frontal gyrus, cingulum, and medial orbital frontal gyrus in the AD group. The findings for these two groups were compatible with previous studies [19], [23]–[24]. In subjects with MCI, some hub regions, such as the medial orbital frontal gyrus, lingual gyrus, and paracentral lobule, were the same as those in the normal controls and in the AD group. These identical hub regions were predominately located in regions of the association cortex, which has been regarded as a pivotal region for receiving convergent information in human cortical networks. The primary location of the hub regions in the association cortex also supports the perspective that it plays a critical role in combining signals from the primary sensory and motor modalities to create emergent psychological properties [30].

#### Changes in nodal centrality

**Figure 5:**
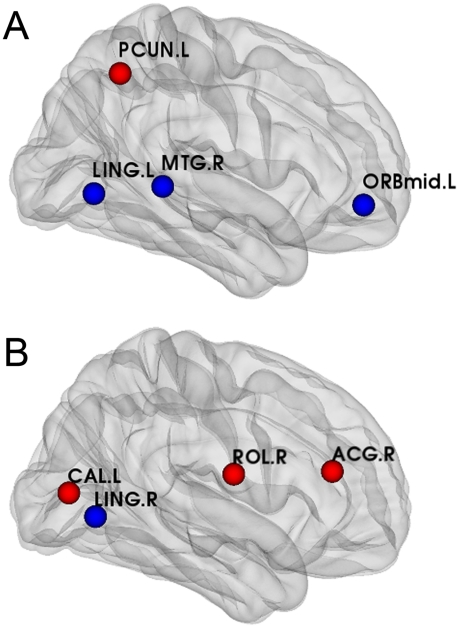
Abnormal changes in between-group nodal centrality in the MCI and AD groups. Each of the eight regions belongs to the hub regions in at least one of the three cortical networks and showed a significant difference (p<0.05). The blue spheres indicate significant decreases in between-group nodal centrality. The red spheres indicate significant increases in between-group nodal centrality. **A** - Abnormal changes shared by the MCI and AD groups. **B** - Abnormal changes only in the AD group. Note that no abnormal changes occurred only in the MCI group. For the abbreviations of the regions, see Table 2.

1000 nonparametric permutation tests were used (see Materials and Methods) to inspect the between-group differences. The regions with significant abnormal changes in nodal centrality in MCI and AD groups are shown in Figure 5. Compared with the NC group, the nodal centrality of the MCI and AD groups significantly decreased in the left lingual gyrus, middle temporal gyrus, middle orbital frontal gyrus and significantly increased in the precuneus. Moreover, compared with the NC and MCI groups, the nodal centrality in AD population showed significant decreases in the right lingual gyrus and significant increases in the right rolandic operculum, anterior cingulum and left calcarine. Additionally, compared with NC and AD groups, no brain areas showed significant changes in nodal centrality in the MCI population.

#### Changes in the correlation coefficients

**Figure 6:**
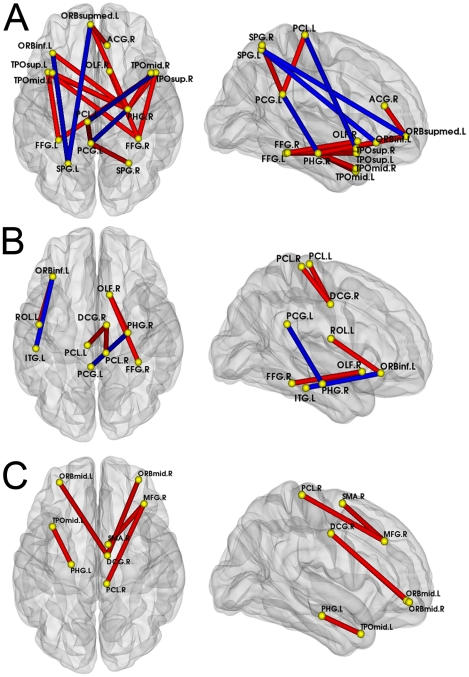
Abnormal interregional correlations in the MCI and AD subjects. The red and blue lines indicate significant between-group differences in interregional correlations between pairs of regions (p<0.01, FDR-corrected); the yellow dots represent those AAL regions with significantly abnormal correlations. The red and blue lines indicate the significantly increased and decreased interregional correlations between the corresponding regions, respectively. **A** - Significant changes in interregional correlations between the NC and AD groups. **B** - Significant changes in interregional correlations between the NC and MCI groups. **C** - Significant changes in interregional correlations between the MCI and AD groups. For the abbreviations of the regions, see Table 2.

Fisher's Z transformation was used to investigate the differences in the between-group interregional correlations (see Materials and Methods). The abnormal interregional correlations that were detected in groups with MCI and AD (p<0.01) are shown in Figure 6. Regions that showed significant changes in the interregional correlations between the NC and AD populations primarily included the parahippocampus gyrus, temporal pole, fusiform, cingulum, superior parietal region and orbital frontal gyrus. The regions that showed significant changes in the interregional correlations between the NC and MCI populations included the parahippocampus gyrus, cingulum, fusiform, orbital frontal gyrus, olfactory, paracentral lobule, inferior temporal gyrus, and rolandic operculum. The regions that showed significant changes in the interregional correlations between the MCI and AD populations included the middle frontal gyrus, superior motor area, paracentral lobule, parahippocampus, temporal pole, orbital frontal gyrus, and middle cingulum. As we can see from Figure 6, our results were consistent with previous studies, which reported progressively increased short distance connectivity and progressively decreased long distance connectivity from MCI to AD [17], [19], [31].

## Discussion

In this study, we constructed cortical networks of NC, MCI and AD groups by calculating correlation coefficients between pairs of gray matter regions. Gray matter, which primarily consists of neuronal cell bodies, is a major component of the central nervous system and can directly reflect the function in the brain. Gray matter volume has been widely adopted as an important measurement by many studies [10], [12], [16], [23], [27], [32]–[34]. Covariation of gray matter volume might provide additional insight into the topographical organization of multiple cortical regions, as indicated by a previous study which reported that related components of the visual system covaried in volume across individuals [22], [27], [34]. Mechelli *et al.* analyzed the level of covariation in gray matter density in cortical regions to investigate brain symmetry [27]. They suggested that covariation might be the result of mutually trophic influences or common experience-related plasticity and that the level of covariation might be disrupted in some patient populations. Raz *et al.* examined hemisphere-related differences in the cerebral cortex using the gray matter volume [34]. Bassett *et al.* constructed a whole-brain anatomical network by compiling a matrix of correlations in gray matter volumes between all pairs of regions [22]. In the present work, we took into account the cortical networks of NC, MCI and AD populations to investigate synthetically the abnormal structure of cortical networks in MCI and AD. For the first time, we investigated the characteristics of cortical networks as an aid in understanding the abnormal structural brain network in subjects with MCI. The main finding of this study was that the characteristics of the cortical network in the MCI populations displayed an intermediate position between those of NC and AD subjects. The relevant detailed attributes of the three cortical networks were: 1. The cortical networks in the NC, MCI and AD groups all showed small-world properties. 2. Abnormal nodal centrality changes were detected in the cortical network in the MCI and AD groups. 3. Significant changes in the interregional correlations were found in populations with MCI and AD. These results may indicate that a loss of small-world characteristics was shown in the cortical network of MCI subjects, as has previously been identified in AD populations. These hub regions and the interregional correlations of the cortical network in MCI provided additional structural evidence to support the opinion that MCI is the transitional stage between normal aging and AD.

### Small-world properties of the three cortical networks

Small-world properties, which are frequently found to be properties of complex networks, seem to be common to a wide variety of information systems. Since gray matter volume has played an important role in brain research in recent years [14], [35]–[36], we constructed cortical networks using gray matter volumes to investigate small-world properties in subjects with MCI and AD. Previous studies have showed abnormal cerebral structures accompanied by atrophy of the gray matter in groups with MCI and AD. Therefore we hypothesized that the small-world characteristics of the cortical network in an MCI population might be the similar to that in AD patients, which was characterized by a higher clustering coefficient and a longer absolute path length [18]. We observed that the clustering coefficient and the absolute path length of the cortical network in subjects with MCI exhibited median values between those of the normal controls and those with AD over a wide range of sparsity threshold values. Using permutation tests, we detected statistically significant differences in the clustering coefficients and absolute path lengths between the normal controls and patients with AD (p<0.05). However, we found no significant difference in the two measurements between the NC and the MCI population and between the MCI and AD populations over the entire range of sparsity threshold values. A previous study indicated that the global gray matter volume in their MCI population was intermediate between that of the normal controls and patients with AD but was not significantly different from either group [14]. Our result suggests that MCI forms a boundary between normal aging and AD. We also found that MCI was not statistically significantly different from either group in the characteristic measurements of cortical networks.

### Altered interregional correlations in MCI and AD

Details of the abnormal interregional correlations in groups with MCI and AD are shown in Figure 6. Compared with the normal controls, the AD patients were found to show a significant increase in their interregional correlations, mainly in the parahippocampal gyrus, temporal pole, fusiform gyrus and cingulum. We also observed that the locations of these involved regions were distributed within a limited area of the whole brain. Our results support recent resting-state fMRI studies which reported increased interregional functional connectivity within each brain lobe and decreased interregional functional connectivity between brain lobes in AD [31]. Decreased interregional correlations in patients with AD, that is, the disappearance of positive correlations between the right parahippocampal gyrus and the posterior cingulum, might suggest impairment of learning and memory [37].The disrupted connectivity between the hippocampus and the posterior cingulum may account for the posterior cingulum hypometabolism that has commonly been detected in positron emission tomography (PET) studies of early AD [38]. Compared with the normal control and AD groups, fewer regions showed significant changes in the interregional correlations in MCI group. Consistent with the concept that MCI is a transition stage in the evolution of AD, subjects with MCI showed the same status as those with AD, that is, increased short distance interregional correlations and decreased long distance interregional correlations. Compared with MCI population, we also observed increased short distance interregional correlations, but no decreased interregional correlation was significant in patients with AD. The abnormal increased interregional correlations may explain the higher clustering coefficients of cortical networks in groups with MCI and AD. Our results suggest that the cortical network structure is seriously abnormal and show a progressive loss of small-world characteristics in subjects with MCI [39].

### Abnormal changes in nodal centrality in MCI and AD

The hub regions of the cortical network that we identified in the normal controls were compatible with those found in previous studies of functional and structural cortical networks [24], [40]–[41]. These hub regions, which are thought to be the substrates for human cognition and consciousness, are in the association cortex that receives convergent inputs from multiple other cortical regions. Previous studies have found that subjects with MCI had a significant reduction in the amount of gray matter in the medial temporal lobe, hippocampus, inferior parietal areas, posterior cingulum, and the lingual and fusiform gyri [13], [33], [42]. In addition, a significant reduction in the gray matter in the frontal cortex has been found in patients with AD [15]–[16]. We expected to find that the structure of the cortical network in groups with MCI and AD changed in these regions along with the atrophy in their gray matter. Our result showed that the middle temporal gyrus, superior temporal pole, lingual gyrus, and parahippocampal gyrus were no longer hubs in groups with MCI and AD. A portion in the these abnormal changes of hub regions in MCI and AD belong to the default mode network, which has been hypothesized as being profoundly relevant to cognitive processing [43]. The abnormal hub regions in the default mode network could result from a decrease in brain metabolism that may occur in the course of the development of AD [38]. Figure 5 shows the regions with abnormal changes in nodal centrality. In fact, these results are to some extent consistent with previous studies. Abnormal changes in the middle temporal gyrus in subjects with MCI and AD were reported as being related to a decline in verbal memory performance [44]. Less activation, as measured using fMRI was detected in the lingual gyrus and cingulate in subjects with MCI and AD [45]. In the present study, the nodal centrality in the precuneus showed a significant difference between the NCs and MCIs and ADs and no significant difference between the MCIs and the ADs. This finding supports a previous study which indicated that differences in the activity in the precuneus were only distinguishable between ADs and NCs, not between the MCI and AD groups [5]. The calcarine and anterior cingulate areas of the cortex seem to be notably spared until the late stages. This sparing of some cortical areas might explain why the nodal centrality of the two areas is abnormal only in patients with AD [32]. From Figure 5, we can see that almost all the brain areas with abnormal changes in nodal centrality showed gradual changes along the transition from NCs to ADs and that no area with abnormal changes was only detected in MCI group. This result also implicates MCI as an intermediate stage between normal aging and AD. The longer absolute path length in subjects with MCI and AD may indicate that the disappearance of these hub regions disrupted the large-scale connections between pairs of brain regions [19], [46]. Meanwhile, we also observed that some regions which had a higher nodal centrality in MCI and AD became new hub regions. Previous studies have reported that increased functional connectivity occurred widely in MCI and AD in various brain regions [17], [47]–[48]. Such increased connectivity may effectively serve as a compensatory system. This compensatory mechanism may play an important role in MCI and AD by enabling patients to use additional cognitive resources to approach a normal level [49]–[51]. The abnormal characteristics of the cortical networks which we observed in MCI and AD may reflect anatomical structural abnormalities. Such a relationship may contribute to an understanding of the cerebral organization in MCI and AD.

### Methodological limitations and perspectives

Our study also has some limitations. Firstly, only small amounts of 3T MRI data are available from the ADNI database. To ascertain the real cortical networks as accurately as possible, this study included as many subjects as were available from each group of the ADNI database, which made the sample size of each group inconsistent. Furthermore, we might have been able to demonstrate this transition from normal aging to MCI to Alzheimer's disease if we had had a larger sample size. As it was, our MCI population could not be separated from the normal controls or from the Alzheimer's group, but the combined groups could clearly be separated from each other. Secondly, Pearson correlation was adopted instead of partial correlation analysis. In fact, after a linear regression of the age, gender and total gray matter volumes of each subject, the matrices of gray matter volumes were not full rank. Thus, a partial correlation analysis could not be performed because the sample size was not large enough. That is why the Pearson correlation was adopted in this work. Finally, compared with the anatomical connectivity obtained by diffusion-based imaging, the method we used in the present study only measures the anatomical connectivity indirectly. However, it is more practical for revealing the anatomical connectivity patterns of the human brain because of its relatively low computational load and simple definition of the neuronal elements (regions) and connections. Future studies should be done to further investigate this issue and replicate our findings using diffusion-based imaging.

In this paper, we only studied the global network manifestation of brain malfunction in MCI and AD based on gray matter volume correlations, an indirect anatomical connectivity. In order to understand the pathophysiological mechanism of MCI and AD, it is necessary to integrate the multi-level network features obtained with various functional and anatomical brain imaging technologies on different scales. On macroscale, such features can be obtained from networks based on illness special region of interest, networks related to special cognitive function, and whole brain networks. Here we would like to propose a concept of brainnetome to represent such integration framework. We define the essential components of brainnetome as network topological structure, performance, dynamics, manifestation of functions and malfunctions of brain on different scales. In fact, a big project (973 program) has been approved in China to conduct studies of brainnetome for four different diseases with focal lesion (stoke and glioma) and diffusion lesions (schizophrenia and AD). For AD, the goal is to find biomarkers on network level which can predict whom of MCIs will develop into AD. We envision that brainnetome will become an emerging co-frontier of brain science, information technology, neurology and psychiatry. Some long-standing issues in neuropsychiatry may be solved by combining brainnetome with genome.

## Materials and Methods

### Subjects

All the subjects used in this study were selected from the Alzheimer's disease Neuroimaging Initiative (ADNI) database (http://www.loni.ucla.edu/ADNI/). This project is the most comprehensive effort to date to identify neuroimaging and other biomarkers of the cognitive changes associated with MCI and AD. The primary goal of this project is to measure the progression of MCI and early AD, in order to develop improved methods for clinical trials in this area. This study included 98 NCs who ranged in age from 70.02 to 90.74 (*M = 77.27*; *SD = 4.66*) (female/male, 49∶49), 113 MCI subjects who ranged in age from 56.28 to 89.40 (*M = 75.12*; *SD = 7.60*) (female/male, 34∶79), and 91 AD subjects who ranged in age from 55.73 to 90.20 (*M = 76.16*; *SD = 7.81*) (female/male 41∶50).

### Image acquisition

The datasets included standard T1-weighted MR images acquired sagittally using volumetric 3D MPRAGE with 1.25×1.25 mm in-plane spatial resolution and 1.2 mm thick sagittal slices. All the high-resolution magnetic resonance images were obtained using 1.5 T scanners. Images using 3T scanners were excluded to remove the discrimination that might be introduced by using different magnetic field strengths. All scans were downloaded in the DICOM format and finally converted to the NIFTI format. Detailed information about the MR acquisition procedures is available at the ADNI website.

### Measure of gray matter volume

All the structural images were preprocessed using voxel based morphometry (VBM) implemented with Statistical Parametric Mapping software (SPM5) running under Matlab 7.0 on the Ubuntu operating system. VBM is a whole-brain, unbiased, semiautomatic, neuroimaging analysis technique that allows the investigation of regional differences in brain volume. In brief, the average gray matter volumes of each brain area were obtained for each subject using the following steps. First, all the structural images were corrected for non-uniformity artifacts. Then, the corrected images were registered to an asymmetric T1-weighted template using nonlinear normalization. Next, the corrected and normalized images were segmented into gray matter, white matter, cerebrospinal fluid and other background classes. Fourth, the resulting gray matter images were smoothed by a 4 mm isotropic Gaussian kernel to compensate for the inexact nature of the spatial normalization. Finally, from these smoothed gray matter images, we calculated the average gray matter volumes for each of n = 90 brain areas, which were comprised of 45 cortical regions in each hemisphere (excluding the cerebellum) in each participant.

### Construction of the structural cortical network

Before obtaining the structural cortical networks, the anatomical connection matrix was calculated. In this study, the structural connections of the cortical network are defined as statistical correlations between pairs of average gray matter volumes from the corresponding AAL areas. We considered that a structural connection existed if the correlation coefficient for a pair of brain areas was statistically significant. Subsequently, an interregional correlation matrix (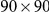) was obtained for each group by calculating the Pearson correlation coefficients across individuals between the average gray matter volumes for each pair of brain areas. We tested the difference in age between the three groups and found a significant difference between the NC and MCI groups (p = 0.016). We found no significant difference for the NC and AD as well as for the AD and MCI in age (p = 0.23 and p = 0.34 respectively). Prior to the correlation analysis, a linear regression was performed to remove the effects of age, gender and total gray matter volume on the full set of individual measurements in each region. The residuals of this regression represented the regional volumes corrected for age, gender and total gray matter volume and provided the substrate for additional analysis. Finally, the matrices for the interregional correlations were obtained with diagonal elements equal to one and the number of total probable connections 90×89/2 = 4005.

### Graph theoretical approaches

Structural cortical networks for each group were represented by binarized matrices P_ij_ with N nodes and K edges. In each case the nodes and the edges corresponded to the AAL areas and the undirected connections between the pairs of AAL areas, respectively. If the same correlations threshold had been applied to the interregional correlation matrices of all the three groups, the topology of the three cortical networks would have differed markedly from each other. In that situation, the resulting graphs would have been comprised of different numbers of edges, even though they were based on the same threshold. Thus, the between-group differences in the three groups would not have done a good job of reflecting alterations in the cortical network topology. To accommodate for this difficulty, sparsity (S) was applied to threshold the interregional correlations matrices of the three cortical networks into binarized matrices. Sparsity is defined as the total number of edges, K, in a graph divided by the maximum possible number of edges. In our case, the maximum possible number of edges equals 90×89/2 = 4005. Since no definitive way for selecting a single threshold value exists, we thresholded each interregional correlations matrix repeatedly over a range of sparsities (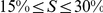) [19]. Utilizing a lower sparsity would not allow for the creation of a fully connected network with 90 nodes, but using a higher sparsity would introduce a lot of spurious edges into each network. Therefore this range of sparsities was chosen because it allows for the creation of fully connected undirected graphs that permit a reasonable estimation of the properties of the graphs. Furthermore, to investigate the abnormal connectivity and hub regions in groups with MCI and AD, a fixed sparsity (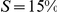), which can minimize the number of spurious edges, was used to construct the cortical networks of the three groups [18], [52]. Using this method the three resulting graphs had the same numbers of nodes and edges.

### Small-world properties analysis

Small-worldness is a ubiquitous property of complex real-life networks that supports both modular and distributed dynamic processing as a principle of brain topology [23]. Certain measurements are usually used to describe small-world properties, such as: mean network clustering coefficient (C_p_) and mean network shortest absolute path length (L_p_). A shorter absolute path length and a higher global efficient may indicate a higher speed of information dissemination and more efficient information processing [29]. In brief, the C_p_ is the average of the clustering coefficient over all the nodes in a network, where the clustering coefficient C_i_ of node i is defined as the number of existing connections among the immediately connected neighbors of the node divided by all their possible connections. C_p_ measures the extent of local cliquishness or local efficiency of information transfer of a network. L_p_ is the average of the mean shortest absolute path length over all nodes in the network, where the mean shortest absolute path length of node i is defined as the total shortest absolute path length between node i and all the others divided by 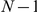. A real network has been found to exhibit small-word characteristics if it meets the following criteria: 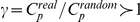 and 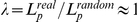, where 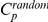 and 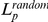 are the mean network clustering coefficient and the mean network absolute shortest path length of matched random networks that have the same number of nodes, edges, and degrees distribution (the degree K_i_ of a node i is the number of connections to the node) as the real network [21].

### Nodal centrality

We investigated the nodal characteristics of the cortical network among the NC, MCI and AD groups. To do this, we introduced the betweenness centrality of the nodes in the networks. Betweenness is a measure of the centrality of a node in a graph. The betweenness, B, of node i is defined as the the number of absolute shortest paths that are between any two other nodes and that run through node i. For further comparison, the betweenness B_i_ would be normalized as 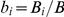, where B is the average betweenness of the network. Based on this concept, the hub nodes that occur on many shortest paths between other nodes have higher betweenness than those that have fewer paths between them. In this study, a node i in which 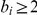 was defined as a hub of the network.

### Statistical analysis

Interregional correlation differences. In order to test whether the interregional correlations of the cortical networks were significantly different among the NC, MCI and AD groups, Fisher's z transformation was applied to convert the correlation coefficients to z values which were approximately normally distributed [53]. A z statistic was used to compare these transformed z values to determine the significance of the between-group differences in the interregional correlations. To correct for multiple comparison, a false discovery rate (FDR) test was performed using a q value of 0.01 [54].

Differences in network topology. We used a nonparametric permutation test to test the statistical significance of the between-group differences in the two characteristics of the cortical networks, C_p_ and L_p_. In this permutation test, we obtained a reference distribution on which we calculated possible values of the test statistic after repeatedly rearranging the observed data from the NC, MCI and AD groups. First, we calculated the C_p_s and L_p_s of the real cortical networks at a given sparsity for each of the three groups separately. To test whether these measurements were significantly different between the three groups, we pooled the data from ADs with the data from the NCs. From this pooled group, we randomly choose some of the subjects to be considered as NCs and the rest to be considered as AD patients. The number of supposed AD patients was equal to the number of actual patients in the original group. We then calculated the differences between the new groups and repeated this process 1000 times. In each of the 1000 cases, we used the same sparsity threshold to generate corresponding binarized matrices and computed the C_p_ and L_p_ characteristics of the two cortical networks for each randomized groups, obtaining the between-group differences. We sorted the 1000 recorded differences and observed whether the between-group differences in the real cortical networks were contained within 95% (two-tailed) of the supposed between-group differences. If they were, we accepted the null hypothesis that the two groups had identical probability distributions at the 5% significance level; otherwise we rejected the null hypothesis. This permutation test procedure was repeated over the range of sparsity threshold values from 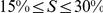. This same procedure was repeated comparing the MCI with the AD groups and comparing the NC with the MCI groups.
